# Role of Scar‐Associated Macrophages in Organ Fibrosis Diseases

**DOI:** 10.1155/jimr/8393673

**Published:** 2026-01-28

**Authors:** Runmin Ding, Zexin Yang, Yuxi Chen, Qiqin Yu, Yisheng Ji, Zijie Wang, Min Gu

**Affiliations:** ^1^ Department of Urology, Jiangsu Key Laboratory of Urological Disease Prevention and Treatment, The Second Affiliated Hospital of Nanjing Medical University, Nanjing Medical University, Nanjing, China, njmu.edu.cn; ^2^ The Second Clinical Medical College, Nanjing Medical University, Nanjing, China, njmu.edu.cn; ^3^ Department of Urology, The First Affiliated Hospital with Nanjing Medical University, Nanjing, China, njmu.edu.cn; ^4^ The First Clinical Medical College, Nanjing Medical University, Nanjing, China, njmu.edu.cn

**Keywords:** macrophage, organ fibrosis, scar-associated macrophages, secreted phosphoprotein 1 (SPP1)

## Abstract

Fibrosis refers to the scarring and hardening of tissue resulting from the excessive deposition of extracellular matrix (ECM) proteins by myofibroblasts during chronic inflammatory responses, which can ultimately lead to organ failure and potentially death. Different states of macrophage activation are critical in regulating both the progression and regression of fibrosis. However, the conventional M1/M2 polarization model fails to accurately capture the dynamic and heterogeneous states of macrophages observed in vivo. As a result, an increasing number of studies have begun to classify macrophages based on their functional phenotypes. One specific subset of functionally distinct macrophages, termed scar‐associated macrophages (SAMs), has been confirmed to play a significant regulatory role in organ fibrosis. This review provides a comprehensive summary of recent advances in understanding the role of SAMs in fibrotic diseases across multiple organs, including the liver, lungs, heart, and kidneys. It further explores the emerging conceptual framework of hot and cold fibrosis, highlighting the potential role of SAMs as central cellular mediators that sustain a positive feedback loop between inflammation and fibrosis in the “hot fibrosis” state. Building on these insights, this review also discusses future research directions aimed at developing targeted therapeutic strategies to modulate SAM activity and mitigate fibrosis in diverse organ systems.

## 1. Introduction

Fibrosis, characterized by the deposition of collagen and other extracellular matrix (ECM) molecules, primarily by fibroblasts, emerges as a pivotal factor contributing to tissue dysfunction in chronic diseases [1]. It can occur in various organs and is recognized as a leading cause of morbidity and mortality worldwide [2]. In developed countries, fibrosis is estimated to account for 45% of deaths [2, 3]. Key regulators of organ fibrosis include inflammatory monocytes and tissue‐resident macrophages [4]. Tissue injury triggers an inflammatory response, leading to recruitment, proliferation, and activation of various immune cells for tissue repair [5].

Among immune cells, macrophages play a crucial role in chronic inflammation and fibrosis, serving as central coordinators of the tissue response to injury [4]. Macrophages are highly plastic and can be polarized into different phenotypes and functions depending on local microenvironmental stimuli, including the phagocytosis of pathogens, infectious debris, and dead cells [6]. The current method for classifying macrophages involves stimulating them in vitro with IFN‐γ/LPS to define M1 macrophages (classically activated), while IL‐4/IL‐13 is used to define M2 macrophages (alternatively activated) [7]. This simplified M1/M2 paradigm was useful in early studies, but in vivo profiling has shown that macrophage gene expression can combine both “M1” and “M2” signatures simultaneously [8]. Consequently, the M1/M2 subtype model does not accurately represent the spectrum of macrophage states, especially in pathological conditions [9, 10]. In contrast, macrophage activation is increasingly seen as a continuum. Computational “archetype” analysis of single‐cell transcriptomes shows that macrophage populations form a continuous low‐dimensional space, with vertices (archetypes) representing specialized functional programs [11–13]. For instance, Miyara et al. [14] applied archetype analysis to macrophages in postinfarction cardiac tissue and delineated a tetrahedral continuum of four conserved functional states: ECM‐remodeling, phagocytic, inflammatory, and metabolic.

As a result, new functional classifications have emerged to replace the classical M1/M2 scheme. One such class of functionally defined macrophages is scar‐associated macrophages (SAMs), which have come to the forefront of fibrosis research [15–23]. SAMs, potentially originating from monocytes, localize to scars and induce myofibroblast activation in response to ecological niches and inflammatory signals that guide their differentiation and function [15, 19, 21, 24–27]. SAMs and their role in maintaining a pro‐fibrotic macrophage niche have garnered significant attention.

This review explores the critical roles and mechanisms of SAMs in organ fibrosis, systematically addressing their identification, distribution, and regulation. By highlighting recent advances, including new conceptual frameworks and experimental findings, we aim to provide insights into SAM formation and propose therapeutic strategies, ultimately offering new perspectives for understanding fibrosis progression and improving clinical treatment.

## 2. Characteristics of SAMs

### 2.1. Identification and Phenotype of SAMs

Previous studies have demonstrated that macrophage polarization plays a decisive role in determining organ fate during inflammation or injury [6, 28]. As key regulators of fibrosis, macrophages undergo significant phenotypic and functional changes following tissue injury [29]. These alterations can lead to maladaptive repair, chronic inflammation, and pathological fibrosis [28].

**Figure 1:**
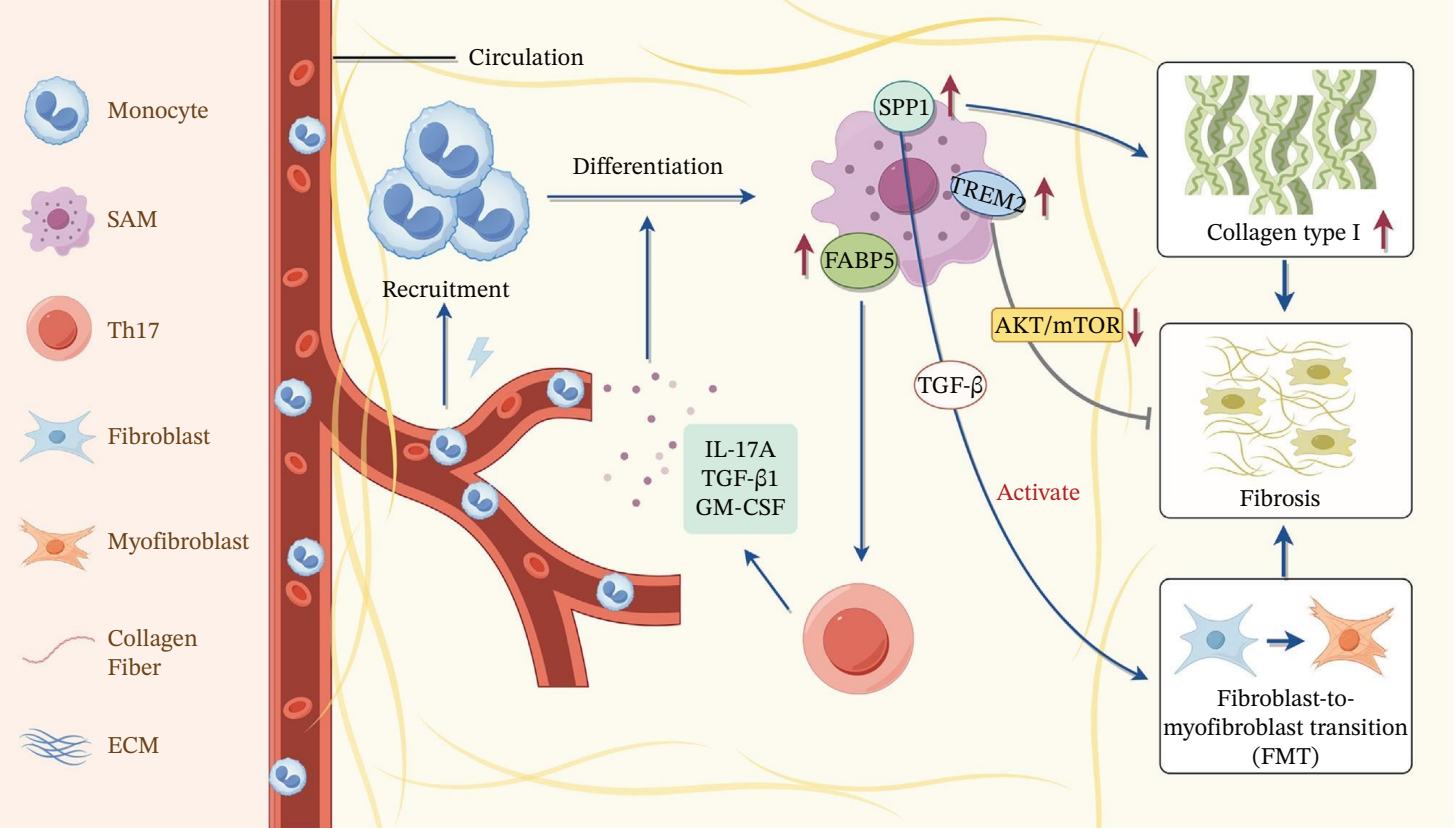
Phenotypes of monocyte‐derived SAMs within fibrotic ecological niches across different tissues. After circulating monocytes are recruited to tissues, they differentiate into SAMs within the fibrotic ecological niche under the influence of signals such as IL‐17 A, TGF‐β1, and GM‐CSF. SAMs highly express *SPP1*, *FABP5*, and *TREM2*. All are pro‐fibrotic except for TREM2, which ameliorates fibrosis. SPP1 protein activates FMT through TGF‐β signaling, thereby promoting fibrosis progression. FABP5 and SPP1 positively regulate Th17 responses, potentially promoting feed‐forward loops that lead to increased levels of IL‐17 A, GM‐CSF, and TGF‐β1.

SAMs, as a pro‐fibrotic macrophage subtype, have significant potential to drive fibrosis progression. According to Fabre et al. [29] SAMs are defined by three criteria: (i) expansion within fibrotic tissue; (ii) proximity to excessive ECM; and (iii) expression of at least five specific markers, including TREM2, CD9, SPP1, GPNMB, and FABP5 (Figure 1). Moreover, they refer to these SAMs as “Fab5 SAMs,” which denotes SAMs positive for five specific markers. SAMs are reported to originate from circulating monocytes and represent a terminally differentiated state within the fibrotic niche [29]. Their differentiation is driven by inflammatory signals: *SPP1* ^*+*^ SAMs can be induced by neutrophils and activated TGF‐β1 (modulated by IL‐17A and GM‐CSF) to promote pathogenic ECM deposition [15].

Various genes associated with fibrosis progression are expressed in Fab5 SAMs, with the *SPP1* gene being of particular concern (Figure 1). Secreted phosphoprotein 1 (SPP1), also known as osteopontin (OPN), has been identified as a highly selective marker of macrophage expansion subpopulations in human multi‐organ fibrosis [16, 20, 30, 31]. SPP1 protein can promote fibrosis progression by inducing fibroblast‐to‐myofibroblast transdifferentiation (FMT) through TGF‐β signaling [32]. Deletion of *SPP1* reduces type I collagen expression, active TGF‐β1 levels, and matrix metalloproteinase‐2 (MMP‐2), thereby ameliorating fibrosis [33]. Other fibrosis‐associated genes, such as *FABP5*, can also positively regulate the T helper 17 (Th17) response in conjunction with *SPP1*, potentially facilitating feed‐forward loops that increase levels of IL‐17 A, GM‐CSF, and TGF‐β1 [34, 35].

Interestingly, the term “scar‐related” does not precisely represent fibrosis. Some studies suggest that TREM2 expressed by Fab5 SAMs may prevent fibrosis, likely due to increased inflammation and cell death, along with decreased clearance of lipids and cellular debris in *Trem2* knockout animals [36–38]. Trem2 deficiency downregulates the Akt/mTOR signaling pathway, which inhibits macrophage survival and promotes apoptosis, while also inducing macrophage polarization via the JAK2‐STAT1/3 pathway, thereby exacerbating fibrosis [39]. Therefore, deletion of *Trem2*, whether globally or in specific tissues or cell types, does not result in a “Fab5‐depleted” phenotype, despite the expression of proteins with anti‐fibrotic functions, such as TREM2. Although proteins with anti‐fibrotic functions, such as TREM2, are expressed, we hypothesize that the overall function of Fab5 SAMs is pro‐fibrotic [29].

### 2.2. Interaction of SAMs With Other Cell Types

**Figure 2:**
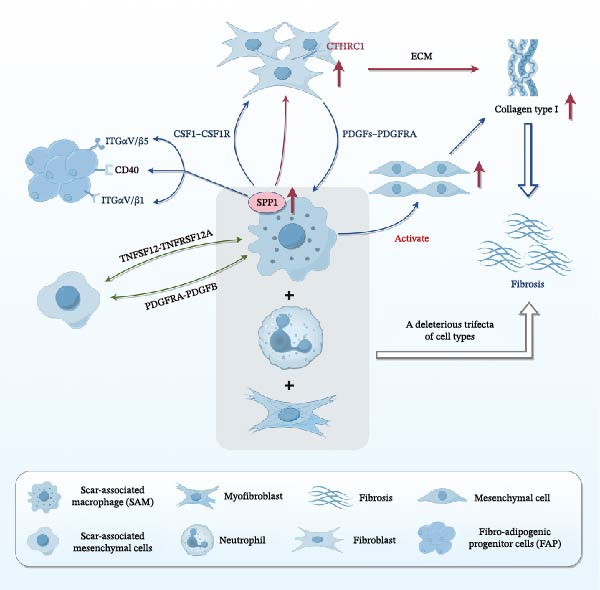
Interaction of SAMs with other cells. SAMs interact with endothelial cells, scar‐associated mesenchymal cells, fibroblasts, FAPs, and other cell types via multiple pro‐fibrotic pathways. SAM‐derived SPP1 signals through CD44 and various integrins to activate fibroblasts. SAMs also form a deleterious trinity with myofibroblasts and *IL-17* ^*+*^ *GM-CSF* ^*+*^ *MMP9* ^*+*^ neutrophils to promote fibrosis.

An increasing body of research suggests that SAMs interact extensively with other cells in the fibrotic niche (Figure 2). Adler et al. [40] provided theoretical support for this idea through a mathematical model of the macrophage–myofibroblast circuit (driven by feedback signals like CSF1 and PDGF). This model predicts that tissue repair can evolve into three possible outcomes: complete healing, a “hot fibrosis” state with persistent, macrophage‐rich inflammation, or a “cold fibrosis” state where myofibroblasts remain active but macrophages are scarce or return to baseline [40]. This “hot vs. cold” classification reflects whether fibrosis is sustained by active immune cell infiltration or primarily by fibroblast autonomous (auto/paracrine) loops [41]. Tissue localization and multi‐omics studies corroborate these concepts. For instance, SAMs are consistently enriched in fibrotic scars alongside myofibroblasts and *IL-17* ^*+*^ *GM-CSF* ^*+*^ *MMP9* ^*+*^ neutrophils, forming a deleterious cellular trinity that secretes multiple pro‐fibrotic factors [29]. Additionally, multi‐lineage ligand–receptor modeling of interactions between SAMs, endothelial cells, and collagen‐producing *PDGFRα* ^*+*^ mesenchymal stromal cells revealed multiple pro‐fibrotic pathways, including TNFRSF12A, PDGFR, and NOTCH signaling pathways, within the scar [15]. Morse et al. [22] demonstrated a causal relationship between *SPP1* ^*+*^ macrophages and fibroblasts by constructing a graphical model showing the direct relationship between differentially expressed genes in *SPP1* ^*+*^ macrophages, fibroblasts, and various other epithelial cell types. They found that the most densely clustered genes were those linking *SPP1* ^*+*^ macrophages and fibroblasts [22]. Cell–cell interaction analysis suggests that *SPP1* ^*+*^ macrophages and fibro‐adipogenic progenitor cells (FAPs) communicate through SPP1‐CD44 and SPP1‐integrin (ITGαV, ITGβ5, ITGβ1) interactions, which promote fibroblast migration and proliferation, thereby contributing to fibrosis in multiple organs [31]. Concurrently, studies have also demonstrated that macrophage‐derived SPP1 enhances ECM expression in *CTHRC1* ^*+*^ fibroblasts, emphasizing the interaction between macrophages and *CTHRC1* ^*+*^ fibroblasts, which further promotes collagen deposition and ultimately contributes to organ fibrosis [22, 42]. Overall, these findings underscore that SAMs engage in complex crosstalk with myofibroblasts and other cell types to regulate fibrotic outcomes.

## 3. SAMs in Organ Fibrotic Diseases

### 3.1. Role of SAMs in Hepatic Fibrosis

The primary factor in the formation of hepatic fibrosis is the excessive deposition and abnormal distribution of ECM components, such as proteoglycans, collagen, and glycoproteins [43]. Immune cells play a crucial role in the development of hepatic fibrosis. Among these, M1/M2 macrophage polarization significantly influences the onset, progression, and outcome of hepatic fibrosis [44]. Macrophages secrete potent inflammatory and pro‐fibrotic factors, including the classic pro‐fibrotic factor TGF‐β1, which promotes myofibroblast activation and ECM synthesis [45].

Both M1 and M2 SAMs are abundantly present in fibrotic livers. However, when hepatic fibrosis improves, M2 SAMs nearly disappear, whereas the number of M1 SAMs remains largely unchanged, and the activity of MMP‐9 is enhanced. This suggests that M1 SAMs play a more significant role in the reversal of hepatic fibrosis [46]. In a mouse model of hepatic fibrosis, SAMs express *SPP1*, *GPNMB*, *FABP5*, and *CD63*. The *SPP1* ^+^ *GPNMB* ^+^ *FABP5* ^+^ subpopulation is a subset of *TREM2* ^+^ *CD9* ^+^ macrophages that is most prominently increased in the human liver and is associated with scarring [29]. Moreover, SAMs have been shown to be present in collagen‐positive scar areas, exhibit a pro‐fibrotic phenotype, and are significantly enlarged in cirrhosis [15]. After chronic liver injury, SAMs accumulate in large numbers near scar tissue and myofibroblasts, which dynamically regulate the activation of hepatic stellate cells (HSCs) and the degradation of ECM. Barnes et al. found that macrophage migration inhibitory factor (MMIF) is essential for the recruitment of SAMs in hepatic fibrosis [47]. In a mouse model of CCL4‐induced hepatic fibrosis, it was shown that MMIF‐deficient mice had reduced SAM recruitment, along with decreased HSC activation and type I collagen production, compared to wild‐type mice. The study further confirmed that reduced SAM recruitment was associated with decreased MMP‐13 expression, suggesting that MMIF recruits SAMs and promotes ECM degradation via MMP‐13, thereby aiding the reversal of hepatic fibrosis [47]. During fibrotic regression, VEGF has been shown to promote monocyte infiltration and SAM accumulation in scar tissue. Interestingly, SAMs, in turn, promote the production of MMP‐13 by Kupffer cells and activated stellate cells through upregulation of *CXCL9* expression, which remodels the scar and ultimately promotes fibrosis regression [48]. Additionally, Plg‐*R*_KT_‐PLG has been identified as a signaling inducer of SAM phenotypic transformation in hepatic fibrosis [49]. Moreover, many growth factors (including VEGF, EGF, and FGF [48, 50]) and key genes (including *CYBA* [51], *AIF1* [52], *CD74* [53], *S100A6* [54], and *C5AR1* [55]) have been reported to promote fibrosis [56]. Ramachandran et al. [15] also identified highly relevant intrascar pathways through which *TREM2* ^+^ *CD9* ^+^ SAMs are induced to promote fibrosis via TNFSF12‐TNFRSF12A and PDGF‐β‐PDGFRα signaling, thereby contributing to hepatic fibrosis. Collectively, these studies identify multiple SAM‐mediated pathways as potential therapeutic targets for anti‐fibrotic intervention in the liver.

### 3.2. Role of SAMs in Pulmonary Fibrosis

Pulmonary fibrosis is characterized by fibroblast proliferation, massive accumulation of ECM, inflammatory damage, increased collagen deposition, and structural destruction of pulmonary tissues, representing a spectrum of end‐stage changes in pulmonary diseases [57, 58]. Macrophages in the lungs, known as pulmonary macrophages, are divided into two main groups: alveolar macrophages (AMs) located in the alveoli and interstitial macrophages (IMs) located in the interstitial tissue of the lungs [59]. Increasing evidence supports the role of AMs and IMs in the progression of PF, suggesting that these macrophages differentiate into M1 or M2 phenotypes depending on the specific microenvironment [60]. In recent years, the role of SAMs in pulmonary fibrosis has gained increasing attention. Reyfman et al. [20] performed single‐cell RNA sequencing (scRNA‐seq) on pulmonary tissue from eight pulmonary fibrosis transplant donors, eight pulmonary fibrosis recipients, and a bronchoscopic cryogenic biopsy sample from patients with idiopathic pulmonary fibrosis, revealing the heterogeneity of AMs and epithelial cells in subjects with pulmonary fibrosis. Three distinct subpopulations of macrophages were identified in both normal and fibrotic lungs: one expressing monocyte markers, one highly expressing *FABP4*, and one highly expressing *SPP1* [22]. Similar to hepatic fibrosis, SAMs are enriched at the periphery of scars and adjacent to activated mesenchymal cells in both human and mouse pulmonary fibrosis. The *SPP1* ^*+*^ *GPNMB* ^*+*^ *FABP5* ^*+*^ subpopulation is the most increased and is closely linked to scarring in human pulmonary fibrosis [29]. Notably, in healthy lungs, these *SPP1* ^*+*^ macrophages exhibit minimal proliferation, but their proliferation rate increases markedly in IPF lungs, indicating that SAM expansion is specifically induced by the fibrotic injury [22]. It has been previously reported that the SPP1 gene is highly expressed in SAMs [15], and that colocalization and causal models support the role of these highly proliferative *SPP1* ^+^ macrophages in myofibroblast activation during pulmonary fibrosis. In bleomycin (BLM)‐induced pulmonary fibrosis, *SPP1* deletion was found to reduce the upregulated expression of type I collagen and MMP‐2 [33, 61]. SPP1 protein can activate FMT through TGF‐β signaling [62, 63]. It was also found that *SPP1* deletion reduced the upregulated expression of type I collagen and MMP‐2 in BLM‐induced pulmonary fibrosis [63, 64]. Serum OPN is elevated in patients with systemic sclerosis [64] and is associated with renal, cardiac, and myelofibrosis, suggesting that *SPP1* ^+^ macrophages may have a broader role in promoting fibrosis. Future research should continue to investigate the specific mechanisms by which SAMs regulate fibrosis.

### 3.3. Role of SAMs in Cardiac Fibrosis

Cardiac fibrosis is characterized by the accumulation of ECM proteins in the cardiac interstitium, leading to systolic and diastolic dysfunction under various cardiac pathophysiological conditions [65, 66]. Coculturing macrophages with cardiac fibroblasts in three‐dimensional peptide gels promotes the expression of α‐smooth muscle actin and collagen in cardiac fibroblasts [67]. It is essential to inhibit the infiltration of nonresident macrophages and promote the proliferation and activation of resident macrophages to ameliorate cardiac fibrosis and improve cardiac function [68]. The specific role of SAMs in cardiac fibrosis remains underexplored. Hoeft et al. found that subclusters of mononuclear phagocyte cells (MPCs) from immune cells identified *SPP1* ^+^ macrophage subsets, where *SPP1*, *APOE*, and *FN1* were specifically expressed. Meanwhile, Studies have confirmed that *SPP1* ^+^ macrophages are the only expanded cluster of macrophages in failing hearts. Furthermore, human cardiac *SPP1* ^+^ macrophages have the highest ECM regulatory factor score among all MPCs, indicating a strong pro‐fibrotic potential in human disease [16]. Shirakawa et al. [69] found that *SPP1* transcriptional activity increased exclusively in *CD206* ^+^ macrophages within the infarcted myocardium, with most CD206^+^ macrophages exhibiting significant *SPP1* transcriptional activation following myocardial infarction, thereby identifying the location and cell type where *SPP1* transcriptional activity is elevated post‐myocardial infarction. Additionally, *SPP1* ^+^ macrophages, which accumulate in perivascular adipose tissue (PVAT) surrounding atherosclerotic coronary arteries, promote the migration and proliferation of fibro‐adipogenic progenitors (FAPs) through SPP1‐CD44 and SPP1‐integrin (ITGαV, ITGβ5, ITGβ1) interactions, thus, aggravating coronary PVAT fibrosis, which is positively correlated with coronary artery stenosis load [31]. Therefore, *SPP1* ^+^ macrophages in coronary PVAT may play a crucial role in the progression of coronary atherosclerosis.

### 3.4. Role of SAMs in Renal Fibrosis

Renal fibrosis represents the final common pathway in nearly all forms of chronic kidney disease (CKD) [70]. Macrophages may promote tissue scarring by recruiting inflammatory cells, secreting growth factors to activate and sustain myofibroblasts, and altering matrix MMP‐mediated ECM homeostasis [24], ultimately leading to renal fibrosis. However, the mechanisms by which macrophages contribute to renal fibrosis in persistent or irreversible kidney injury are not fully understood. Furthermore, the role of SAMs in influencing renal fibrosis has been poorly studied. In a recent study, Hoeft et al. identified a cluster of macrophages (*SPP1* ^+^ macrophages) characterized by the specific expression of *SPP1* and *APOE*. Importantly, *SPP1* ^+^ macrophages were more expanded in CKD kidneys than any other MPC cell cluster [16]. SPP1/OPN, a macrophage chemoattractant [71, 72], is primarily produced by the distal nephron and secreted into urine in the normal kidney [73]. The upregulation of *SPP1* in nephropathy is associated with renal tubulointerstitial fibrosis [71, 74]. Persy et al. [75] found that *SPP1* facilitates the recruitment of macrophages to the kidneys following ischemia and stimulates the development of renal fibrosis after acute ischemic injury. In a tissue microarray study of 41 human kidneys, it was confirmed that the expression of *SPP1* in *CD68* ^+^ macrophages is closely correlated with the expression of collagen type I alpha 1 (COL1A1) in human kidneys, suggesting a potential target for anti‐renal fibrosis therapy. Moreover, cardiac *SPP1* ^+^ macrophages showed the greatest similarity to renal *SPP1* ^+^ macrophages, highlighting a conserved phenotype of macrophage activation across fibrotic organs [16]. Therefore, in‐depth study of the role of SAMs in renal fibrosis may enhance our understanding of the pathological mechanisms underlying renal fibrosis and provide a theoretical basis for the development of novel therapeutic strategies.

### 3.5. Role of SAMs in Other Organ Fibrosis

Furthermore, SAMs play a role in fibrosis not only in the major organs previously mentioned but also in other organs (Table 1). For instance, in dermal fibrosis, macrophage‐produced SPP1 increases ECM expression in *CTHRC1* ^*+*^ fibroblasts, further enhancing the interaction between macrophages and *CTHRC1* ^*+*^ fibroblasts, and promoting collagen deposition [42]. In degenerative ascending aortic aneurysm (AscAA), the expression of *SPP1* in the aortic intima‐media is correlated with the level of macrophage infiltration. Moreover, *SPP1* has been identified as a key gene for fibrotic endothelial‐to‐mesenchymal transition (EndMT) in patients with degenerative AscAA, with ETS1 as a potential regulator of *SPP1* expression [79]. In choroidal neovascularization (CNV), Droho et al. [78] identified *SPP1* ^*+*^ *CD11c* ^*+*^ macrophages in mouse eyes using scRNA‐seq. These *SPP1* ^*+*^ *CD11c* ^*+*^ macrophages stimulate angiogenesis, which is necessary for laser‐induced CNV, and express transcriptomes that promote glycolysis, lipolytic metabolism, and angiogenesis through various VEGF‐dependent and VEGF‐independent pathways [78].

**Table 1:** Evidence of SAMs in organ fibrosis.

Diseases	Patients/animal model	Conclusions	References
SAMs in hepatic fibrosis	Rodent carbon tetrachloride models	SAMs are a major source of MMP13 in resolving hepatic fibrosis	Fallowfield et al. [76]
SAMs in hepatic fibrosis	Models of mice with CCl4‐induced fibrosis	SAMs can upregulate *CXCL9* expression, and *CXCL9* promotes Kupffer and activated stellate cells to produce MMP13 to remodel scar and promote fibrosis resolution	Yang et al. [48]
SAMs in hepatic fibrosis	Models of mice with CCl4‐induced fibrosis	MIF‐dependent recruitment of SAMs contributes to degradation of ECM via MMP13	Barnes et al. [47]
SAMs in hepatic fibrosis	Mouse liver injury models	Plg‐*R*_KT_‐ PLG plays an important role in hepatic fibrosis by mediating SAM transformation	Yang et al. [49]
SAMs in hepatic fibrosis	Noncirrhotic (*n* = 7) and cirrhotic (*n* = 5) human liver tissue	Stromal cell‐secreted IL‐6 limits the macrophage differentiation into *CD9* ^+^ cirrhotic macrophages	Buonomo et al. [77]
SAMs in hepatic and pulmonary fibrosis	Murine models of hepatic and pulmonary fibrosis	SAMs, arising from monocytes and differentiated by IL‐17A and GM‐CSF produced by neutrophils and active TGF‐β1 in the niche, promote the deposition of pathogenic ECM by contributing to the activation of mesenchymal cells and clearing normal ECM	Fabre et al. [29]
SPP1^+^ macrophages in pulmonary fibrosis	Models of murine BLM‐induced pulmonary fibrosis	Inhibition of the interaction of OPN with αv integrin by administration of RMV‐7 antibody improved pulmonary fibrosis in BLM‐treated mice	Takahashi et al. [61]
SPP1^+^ macrophages in pulmonary fibrosis	Models of murine BLM‐induced pulmonary fibrosis	Deletion of SPP1 reduces upregulated expression of collagen type 1 and MMP2 in BLM‐induced lung fibrosis. SPP1hi macrophages upregulated *DC-SIGN* (*CD209*), *LGMN* and *CHI3L1*, all regulated by IL‐4 and/or IL‐13	Morse et al. [22]
SPP1^+^ macrophages in cardiac and kidney fibrosis	Models of *Cxcl4* ^*-/*-^ (C57BL/6‐tm [Cxcl4]) mice	Platelets, the most abundant source of CXCL4 in vivo, control SPP1^+^ macrophage activation via CXCL4	Hoeft et al. [16]
SAMs in CNV	Laser‐induced CNV mouse model of nAMD	SPP1^+^CD11*c* ^+^ macrophages are proangiogenic and necessary for CNV	Droho et al. [78]
SPP1^+^ macrophages in skin fibrosis	Six keloid patients	SPP1 that was principally produced by macrophages increased ECM expression from CTHRC1^+^ fibroblasts	Liu et al. [42]

*Note:* CXCL, chemokine (C‐X‐C motif) ligand; RMV‐7, antimouse αv integrin monoclonal antibody.

Abbreviations: CHI3L1, chitinase 3‐like protein 1; CTHRC1, collagen triple helix repeat containing 1; SPP1, secreted phosphoprotein 1; BLM, bleomycin; CNV, choroidal neovascularization; DC‐SIGN, dendritic cell‐specific intercellular adhesion molecule‐3‐grabbing nonintergrin; ECM, extracellular matrix; GM‐CSF, granulocyte‐macrophage colony‐stimulating factor; IL, interleukin; LGMN: legumain; MIF, migration inhibitory factor; MMP, matrix metalloproteinase; nAMD, neovascular age‐related macular degeneration; OPN, osteopontin; SAMs: Scar‐associated macrophages; TGF‐β1: transforming growth factor‐β1.

## 4. A Novel Immunological Paradigm for Fibrotic Lesions: Stratifying Hot and Cold Fibrosis

Recently, the conceptual framework of “hot fibrosis” and “cold fibrosis” has gained consensus across studies of organ fibrosis. Hot fibrosis refers to fibrotic lesions characterized by sustained immune activation, persistent infiltration of immune cells (e.g., macrophages and T cells), and reinforcement of fibrosis through intercellular positive feedback loops. In contrast, cold fibrosis is marked by immune exclusion and local immunosuppression, where fibrosis is primarily maintained by fibroblasts through autocrine signaling mechanisms. This cold phenotype is commonly observed in late‐stage, stabilized scar tissue [41].

Adler et al. [40] developed a mathematical model that elegantly captured this dual‐outcome framework by simulating the dynamic interplay between recruited macrophages and activated fibroblasts following tissue injury. Notably, this model was among the first to predict two distinct fibrotic trajectories: (i) hot fibrosis, in which SAMs and myofibroblasts persist and reinforce each other through sustained crosstalk; and (ii) cold fibrosis, where myofibroblasts remain, but SAMs diminish, and the macrophage compartment returns to a homeostatic, nonfibrotic state [12, 40].

**Figure 3:**
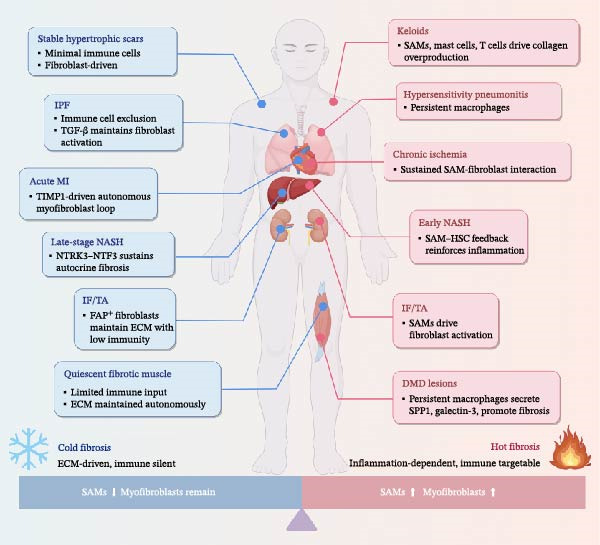
Immuno‐fibrotic spectrum of “hot” and “cold” fibrosis across organs. This illustration depicts representative examples of hot fibrosis (right, red) and cold fibrosis (left, blue) across multiple tissues, highlighting the immune and stromal mechanisms sustaining each fibrotic state. Hot fibrosis is defined by persistent immune activation, particularly sustained infiltration of SAMs, and active crosstalk between immune cells and fibroblasts. This results in reinforcement of fibrogenesis through positive feedback loops involving cytokines and growth factors such as IL‐6, TGF‐β1, and galectin‐3. Hot fibrotic lesions are found in keloids, hypersensitivity pneumonitis, chronic ischemia, early‐stage NASH, inflammatory zones of IF/TA, and DMD lesions. These states are potentially reversible and responsive to immune‐targeted therapies. In contrast, cold fibrosis is characterized by minimal immune involvement, with fibrosis maintained autonomously by myofibroblasts through autocrine and paracrine loops, independent of ongoing immune cell input. This state is typical of stable hypertrophic scars, IPF, acute MI, late‐stage NASH, quiescent fibrotic muscle, and immune‐silent zones of IF/TA. The diagram clearly illustrates the dynamic relationship between SAMs and myofibroblasts in the two fibrotic states: in hot fibrosis, both cell types reinforce each other through sustained interactions; whereas in cold fibrosis, SAMs are reduced or absent, and myofibroblasts ECM accumulation through stable, self‐sustaining autocrine loops.

These predictions have been supported by experimental evidence across organs, including the liver, lung, heart, kidney, and so on, highlighting the relevance of this paradigm (Figure 3). In chronic liver diseases such as nonalcoholic steatohepatitis (NASH), fibrosis often begins in a “hot” state characterized by dense infiltration of SAMs, which engage in sustained crosstalk with HSCs to reinforce a pro‐fibrotic inflammatory feedback loop. As the disease advances, immune cell presence diminishes in certain lesions, and inflammatory cues progressively subside. During this transition, HSCs undergo morphological remodeling and extend cellular projections that facilitate the formation of dense networks of short‐range ligand–receptor interactions. These interactions establish autocrine and paracrine signaling circuits that sustain HSC activation independently of immune input, marking the onset of a “cold fibrosis” state [80]. In a murine model of NASH, Wang et al. [80] demonstrated that targeted inhibition of a key autocrine circuit—the NTRK3–NTF3 signaling axis—effectively disrupted this self‐perpetuating fibrotic program and led to significant regression of advanced fibrosis. These findings provide mechanistic validation for the transition from immune‐dependent to macrophage‐independent, autonomous fibrosis [80]. In the heart, recent works by Miyara et al. [14] applied this paradigm to cardiac injury. They found that acute myocardial infarction predominantly induces a cold fibrosis state, driven by autonomous myofibroblast loops. Conversely, chronic cardiac injury (e.g., repetitive ischemia) leads to a macrophage‐rich hot fibrosis phenotype with sustained SAM–fibroblast interactions. In their murine model, neutralization of the myofibroblast autocrine factor TIMP1 reduced cold fibrosis, highlighting fibrosis type–specific therapeutic opportunities. These findings suggest that the presence of cardiac SAMs depends on the context of injury: SAMs may be transient in acute lesions but persist in response to chronic stress [14]. Pulmonary fibrosis represents a canonical model of cold fibrosis, particularly in IPF. IPF lesions are largely devoid of active immune infiltrates, and fibroblast activation is maintained through autocrine TGF‐β signaling. By contrast, in conditions such as hypersensitivity pneumonitis, macrophages persist within lesions, conferring a partially hot phenotype that remains partially responsive to corticosteroids and other anti‐inflammatory therapies [41]. Similarly, in transplanted kidney interstitial fibrosis and tubular atrophy (IF/TA), fibrotic lesions can manifest as either “hot” or “cold” zones within the same kidney, reflecting local variation in macrophage dependence: some regions harbor ongoing SAM infiltration that drives myofibroblast activity, whereas adjacent areas contain isolated clusters of activated fibroblasts (such as *FAP* ^*+*^ myofibroblasts) that continue producing ECM with minimal immune cell presence [81]. In the skin, keloids are considered prototypical hot fibrotic lesions. They exhibit sustained activation of macrophages, mast cells, and T cells, which release inflammatory mediators such as IL‐6 and TGF‐β1, promoting fibroblast proliferation and excessive collagen deposition. In contrast, hypertrophic or mature stable scars are characterized by minimal immune cell infiltration and a quiescent inflammatory environment, aligning more closely with the cold fibrosis phenotype [82]. In skeletal muscle fibrosis, particularly in diseases such as Duchenne muscular dystrophy (DMD), macrophages are found to persist in damaged muscle tissue. These cells secrete pro‐fibrotic mediators such as galectin‐3 and SPP1, which drive the activation of fibroblast‐like cells and ECM accumulation. These immune‐mediated processes reflect a hot fibrotic environment, suggesting that chronic inflammatory microenvironments in muscle can similarly initiate immune‐dependent scarring [83].

Collectively, hot and cold fibrosis represent a cross‐tissue immuno‐fibrotic spectrum, rather than organ‐specific phenomena. Hot fibrosis often implies an immunologically active and potentially reversible state, offering therapeutic opportunities targeting immune pathways. In contrast, cold fibrosis generally reflects a late‐stage or stabilized scar, requiring strategies aimed at fibroblast function and their microenvironment. Although emerging studies suggest that SAMs may persist in hot fibrotic states, systematic longitudinal data remain lacking. It is thus unclear whether SAMs represent transient injury‐induced responses or chronic fibrosis‐driving populations. Future efforts to classify fibrosis based on immune activity may facilitate more precise stratification and individualized therapeutic interventions across organs and disease stages.

## 5. Conclusions and Prospectives

In this review, we summarize the definitions, phenotypes, and characteristics of recently identified SAMs, as well as their significant role in the progression of organ fibrosis. First, the discovery of SAMs has shifted our understanding from the traditional M1/M2 macrophage polarization paradigm to a more nuanced, functionally defined macrophage classification. Different macrophage activation states play crucial roles in both the progression and regression of fibrosis. Identifying key macrophage populations in human fibrotic tissues may thus lead to new anti‐fibrotic therapies, making the recognition of SAMs particularly significant in the fibrosis field. Secondly, there are striking commonalities in the role of SAMs across different organs and their related fibrogenic mechanisms. SAMs predominantly originate from infiltrating monocytes and differentiate under the influence of pro‐fibrotic signals such as IL‐17A and GM‐CSF produced by neutrophils, along with active TGF‐β1 in the fibrotic niche. These macrophages promote the deposition of pathogenic ECM by activating mesenchymal stromal cells and concurrently contribute to matrix remodeling by degrading normal ECM components. Additionally, SAMs interact with various cell types, including endothelial cells, scar‐associated mesenchymal cells, fibroblasts, myofibroblasts, and FAPs, through multiple pro‐fibrotic signaling pathways. These findings collectively suggest that SAMs play a central role in shaping the fibrotic immune microenvironment in diverse tissues. Thirdly, an emerging conceptual framework now further refines our understanding by delineating two stable fibrosis states based on immune involvement: “hot” fibrosis vs. “cold” fibrosis. The core difference between these states lies in whether fibrosis maintenance relies on continued macrophage‐mediated immune support. This hot‐vs.‐cold paradigm underscores a critical point: the presence of SAMs is indispensable for sustaining a hot fibrotic scar, while in cold fibrosis the scar endures with little to no ongoing macrophage involvement. Finally, looking forward, this conceptual dichotomy of hot and cold fibrosis provides a promising roadmap for stratifying fibrotic diseases and developing targeted treatments. Fibrosis should no longer be viewed as a monolithic process; instead, assessing the degree of SAM infiltration and activity could become an integral step in fibrosis evaluation. Future research should leverage high‐resolution technologies, such as scRNA‐seq and spatial transcriptomics, to classify fibrotic lesions by their immune activity status, distinguishing hot from cold scars in patients. By integrating such stratification into preclinical and clinical studies, investigators can design precision therapies tailored to the fibrosis state: for instance, pairing patients with hot fibrosis to trials of immunomodulatory or macrophage‐targeted treatments and directing those with cold fibrosis toward therapies that inhibit myofibroblast autocrine circuits or enhance matrix resorption. This state‐specific therapeutic approach, supported by the hot/cold framework, could greatly improve treatment efficacy and outcomes in organ fibrosis.

In summary, SAMs have emerged as key drivers of fibrosis, and understanding whether a fibrotic lesion is sustained by these immune cells or has become self‐sufficient is crucial. Embracing the hot vs. cold fibrosis paradigm will not only refine our mechanistic insight into fibrogenesis but also pave the way for innovative, targeted anti‐fibrotic strategies that are tailored to the immunological state of the scar. Such future efforts to align fibrosis therapies with the underlying scar biology hold great promise for improving patient care across a spectrum of chronic fibrotic diseases.

## Author Contributions

Min Gu and Zijie Wang conceived the study and supervised the overall work. Runmin Ding, Zexin Yang, and Yuxi Chen performed literature analysis, drafted and revised the manuscript. Qiqin Yu and Yisheng Ji contributed to the collection and organization of relevant literature.

## Funding

This study was funded by the National Natural Science Foundation of China (Grants numbers 82470790, 82270790, 82170769, 82070769, and 81900684), the Special Fund for Science and Technology Program of Jiangsu Province (Key Research and Development Plan for Social Development Project) (Grant number BE2023784), the Jiangsu Province Natural Science Foundation Program (Grant number BK20191063), and the Postgraduate Research & Practice Innovation Program of Jiangsu Province (Grant number SJCX25_0803).

## Disclosure

All authors are responsible for the integrity of the work as a whole and have approved the final version for publication.

## Conflicts of Interest

The authors declare no conflicts of interest.

## Data Availability

The data that support the findings of this study are available upon reasonable request from the corresponding author.
